# Cystinosis and Cellular Energy Failure: Mitochondria at the Crossroads

**DOI:** 10.3390/ijms27020630

**Published:** 2026-01-08

**Authors:** Francesco Bellomo, Domenico De Rasmo

**Affiliations:** 1 Nephrology Research Unit, Bambino Gesù Children’s Hospital, IRCCS, 00165 Rome, Italy; 2 Institute of Biomembrane, Bioenergetics and Molecular Biotechnology (IBIOM), National Research Council (CNR), 70124 Bari, Italy

**Keywords:** cystinosis, lysosomal storage diseases, mitochondria, bioenergetics, cAMP, mitophagy, cysteamine, flavonoids, ketogenic diet

## Abstract

Cystinosis is a rare lysosomal storage disorder characterized by defective cystine transport and progressive multi-organ damage, with the kidney being the primary site of pathology. In addition to the traditional perspective on lysosomal dysfunction, recent studies have demonstrated that cystinosis exerts a substantial impact on cellular energy metabolism, with a particular emphasis on oxidative pathways. Mitochondria, the central hub of ATP production, exhibit structural abnormalities, impaired oxidative phosphorylation, and increased reactive oxygen species. These factors contribute to proximal tubular cell failure and systemic complications. This review highlights the critical role of energy metabolism in cystinosis and supports the emerging idea of organelle communication. A mounting body of evidence points to a robust functional and physical association between lysosomes and mitochondria, facilitated by membrane contact sites, vesicular trafficking, and signaling networks that modulate nutrient sensing, autophagy, and redox balance. Disruption of these interactions in cystinosis leads to defective mitophagy, accumulation of damaged mitochondria, and exacerbation of oxidative stress, creating a vicious cycle of energy failure and cellular injury. A comprehensive understanding of these mechanisms has the potential to reveal novel therapeutic avenues that extend beyond the scope of cysteamine, encompassing strategies that target mitochondrial health, enhance autophagy, and restore lysosome–mitochondria communication.

## 1. Introduction

Cystinosis is a genetic metabolic disorder classified as a lysosomal storage disease (LSD). It is caused by a mutation in the *CTNS* gene that encodes cystinosin, a lysosomal cystine transporter responsible for exporting cystine from the lysosomal lumen to the cytosol. The most frequent and severe form of cystinosis is nephropathic cystinosis (OMIM 219800), which is diagnosed as growth retardation associated with renal Fanconi syndrome beginning within the first year of life and confirmed by a genetic test [1,2]. The current treatment for cystinosis involves using cysteamine to reduce cystine accumulation in cells, which can significantly improve kidney function and overall health in affected individuals. Depending on the age at which treatment is initiated and the intrinsic vulnerability of each tissue, many extrarenal complications can develop, including those affecting the eyes, thyroid, gastrointestinal apparatus, skeletal apparatus, and neuromuscular system [3].

Intra-lysosomal cystine accumulation, caused by cystinosin transporter defects, is the main characteristic of cystinosis [4]. However, a whole series of cellular processes, such as autophagy, apoptosis, vesicular trafficking, and the redox state, are altered in response or in an attempt by the cell to restore equilibrium. While all these processes are vital for cellular survival, altered energy metabolism could be crucial for the pathological variability in different tissue districts due to their distinct energy profiles [5,6,7,8]. Catabolic and anabolic processes are intricately linked to lysosomes and mitochondria, which serve as recycling centers, energy providers, and key regulators of cellular metabolic activity. These organelles engage in both anterograde and retrograde communication with the cell, thereby influencing the metabolic balance in multiple directions [9,10].

This review highlights the critical contribution of energy metabolism to cystinosis, with a particular emphasis on the oxidative pathways that maintain cellular homeostasis. Mitochondria are increasingly recognized as metabolic powerhouses, as well as key receivers, integrators, and transmitters of environmental and intracellular cues. They function as central signaling hubs that coordinate cellular homeostasis, stress responses, and survival pathways by communicating constantly with other organelles, especially lysosomes, the endoplasmic reticulum, and peroxisomes. As discussed in recent reviews [11,12], this conceptual framework is particularly relevant to cystinosis, in which lysosomal dysfunction disrupts multiple mitochondrial signaling pathways, including nutrient sensing, autophagy, and redox balance.

## 2. The Mitochondria–Lysosome Crosstalk

The compartmentalization of biological membranes within cellular organelles, such as lysosomes, mitochondria, the endoplasmic reticulum, the Golgi apparatus, peroxisomes, and the nucleus, is an evolutionary strategy that allows for the specific execution of biochemical reactions and the organization of different functions. Although traditional concepts considered all organelles inside eukaryotic cells to be independent structures that do not interact with each other, it is now well established that intracellular membrane compartments interact directly. These interactions occur through membrane contact sites (MCSs) [13,14], by proteins such as STARD3 and DMT1 [15], or indirectly via vesicular transport and molecular signals [16,17,18]. Cross-organelle signaling, which involves the transfer of metabolites, lipids, and proteins, is essential for cell function and survival. Mitochondria and lysosomes are highly organized organelles that adopt various strategies to communicate their functional status with each other and the cellular environment [19,20,21]. Interestingly, their position, relative to the plasma membrane and the nucleus, represents a snapshot of the metabolic and redox state. Mitochondria are dynamic organelles that differ in size, shape, and location. Under normal conditions, they spread around the cell relatively uniformly, but in stress and/or pathological conditions, they reposition themselves in perinuclear clustering [22]. The spatial organization of lysosomes is much more complex. They often cluster in the perinuclear region surrounding the microtubule-organizing center (MTOC) in nonpolarized mammalian cells [23], but they also spread into the cell’s periphery. This distribution can be easily altered by changes in cellular nutrient status [24], lysosomal acidification [25], and misfolded protein accumulation [26,27].

Various patterns of lysosome distribution have been described in cystinosis. In some cases, lysosomes are localized to the perinuclear region of proximal tubular cells. This organization may be due to a failure to anchor at the cell periphery [28] or an alteration in autophagic processes [29,30,31]. In other cases, lysosomes are distributed as punctate dispersed structures throughout cystinotic fibroblasts, which is associated with a chaperone-mediated autophagy (CMA) defect linked to the downregulation of the Rab11- and Rab7-effector RILP [32,33].

**Figure 1:**
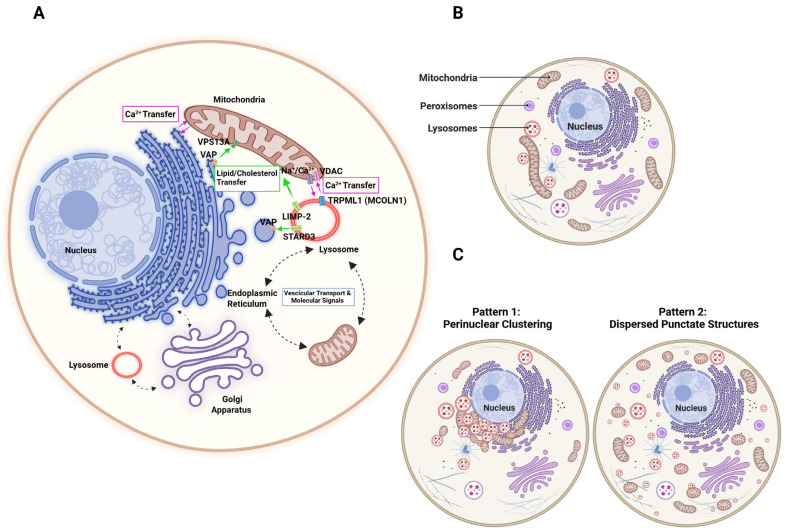
Cellular Organelle Interactions and Distribution Patterns. (**A**) Cross-organelle communication through the exchange of metabolites, lipids, and proteins is critical for cellular homeostasis. Mitochondria and lysosomes establish dynamic contact sites and signaling pathways to coordinate their functional states with each other and the broader cellular environment. (**B**) Organelle distribution in normal conditions. (**C**) Organelle distribution in cystinosis. Abbreviations: VPS13A (Vacuolar protein sorting-associated protein 13); VAP (VAMP-associated protein); VDAC (Voltage-dependent anion channel); TRPML1 (Transient receptor potential mucolipin 1); LIMP-2 (Lysosomal integral membrane protein 2); STARD3 (StAR-related lipid transfer). Created in BioRender. Bellomo, F. (2026) https://BioRender.com/ttym66b.

Mitochondria and lysosomes both interact with the endoplasmic reticulum (ER), and these contacts are critical for the transfer of calcium (Ca^2+^) from the ER to both organelles, as well as for the transfer of phospholipids and cholesterol by the Vps13 protein family [34,35,36]. The recently emerged role of mitochondria–lysosome contacts in regulating mitochondrial Ca^2+^ dynamics has been attributed to the transient receptor potential mucolipin 1 (TRPML1), a lysosomal calcium efflux channel [20]. Several diseases resulting from mitochondrial and/or lysosomal dysfunction also exhibit dysregulation of cellular calcium [37,38,39,40]. Ivanova et al. found no difference in lysosomal Ca^2+^ content or the size of thapsigargin-sensitive Ca^2+^ stores (independent of ER Ca^2+^ stores) between control and cystinotic proximal tubular epithelial cells (PTECs). However, no information was available regarding the Ca^2+^ content of vesicles, such as recycling endosomes and autophagosomes [41]. Alteration of phospholipids and/or cholesterol has not yet been described in cystinotic cells; however, higher cystine levels have been observed in the tissues of the BALB/c mouse model of Niemann–Pick disease type C. In particular, subcellular fractionation of liver homogenates has revealed that cystine accumulates preferentially in the lysosomal fraction [42]. This aspect has never been considered in relation to cystinosis. However, it would be interesting to evaluate the severity of the disease based on cholesterol and lipid imbalances (Figure 1).

## 3. Mitochondria and Cystinosis

The first description of mitochondrial involvement in cystinosis was reported in 1986, when electron microscopy of renal biopsies revealed abnormal mitochondrial ultrastructure [43]. Subsequent studies, however, yielded inconsistent findings, largely due to differences in model systems and experimental approaches. In the 1990s, cystine dimethyl ester (CDME) was used to mimic lysosomal cystine accumulation; although CDME treatment led to ATP depletion, it was later shown that this effect resulted from direct mitochondrial toxicity, rendering CDME an inadequate model of cystinosin deficiency [44,45,46,47].

Energy metabolism was later examined in primary fibroblasts from cystinosis patients, which showed reduced ATP levels compared with controls [48,49]. Similar results were observed in rabbit proximal tubular cells with cystinosin silencing [50], although clear mitochondrial structural defects were not detected in these early studies.

More recent investigations have consistently reported a range of mitochondrial abnormalities, including reduced respiratory chain (RC) activity and membrane potential, altered mitochondrial dynamics and structure, and increased mitophagy [29,51,52,53,54,55,56]. These defects have been associated with dysregulated signaling pathways, such as alterations in cAMP in conditionally immortalized proximal tubular epithelial cells [53] and perturbations in mTOR signaling [57]. Additional evidence for energy imbalance includes activation of AMP activated protein kinase (AMPK) in primary rabbit PTECs with cystinosin knockdown and inhibition of pyruvate kinase and creatine kinase activities in tissues from cystine-treated rats [58,59,60,61].

Apparent discrepancies across studies can now be reconciled by considering differences in experimental models, methodologies, and the cell type specificity of mitochondrial vulnerability. CDME based models produced artefactual mitochondrial toxicity, explaining why early observations were difficult to integrate with later findings. Fibroblast based studies, while demonstrating reduced ATP, failed to reveal pronounced mitochondrial or autophagy defects—likely reflecting the lower metabolic demand of fibroblasts compared with proximal tubular epithelial cells, which depend heavily on oxidative phosphorylation and robust mitochondrial quality control mechanisms.

In contrast, work in *Ctns*^−/−^ proximal tubular cells and zebrafish models has consistently revealed impaired clearance of damaged mitochondria via mitophagy, a defect not observed in *Ctns*^−/−^ fibroblasts. Collectively, these results indicate that mitochondrial dysfunction in cystinosis is highly cell-type specific, with the most pronounced abnormalities occurring in metabolically active and autophagy dependent proximal tubular epithelial cells. Incorporating this perspective reconciles earlier conflicting findings and provides a coherent mechanistic framework for how lysosomal cystine accumulation drives mitochondrial impairment.

### 3.1. Mitochondrial Oxidative Phosphorylation System (OXPHOS)

The OXPHOS includes mitochondrial RC complexes and the F_1_F_O_-ATP synthase. The RC consists of NADH-ubiquinone oxidoreductase (complex I), succinate-ubiquinone oxidoreductase (complex II), ubiquinone-cytochrome c oxidoreductase (complex III), and cytochrome c oxidase (complex IV). The proteins of the OXPHOS are encoded by nuclear and mitochondrial DNAs [62,63].

The first observation of altered mitochondrial RC dysfunction revealed a reduction in mitochondrial ATP levels when using a complex I substrate (malate-glutamate) to fuel the RC in human renal tubular cystinotic epithelial cells [51]. This did not occur with the complex II substrate (succinate), indicating a possible complex I deficiency. This finding was reproduced in a *Ctns* knockout mouse model, which showed a reduction in complex I substrate-dependent respiration, associated with a proton leak and decreased ATP production [29]. Confirmation of complex I activity impairment, as measured by V(max) activity, was demonstrated in conditionally immortalized proximal tubular epithelial cells from two patients with different *CTNS* gene mutations and in human kidney-2 (HK-2) cells silenced for the *CTNS* gene [53]. Altered mitochondrial complex I activity in cystinosis may be associated with the emerging “dynamic assembly” theory of complex I [64]. This hypothetical mechanism suggests that, once imported into mitochondria, several peripheral subunits of complex I replace pre-existing, oxidized, aged copies that are already assembled into the complex, thereby modulating complex I activity [65]. Regarding this theory, a specific downregulation of the complex I subunits involved in dynamic assembly has been found in cystinotic cells [53], suggesting a possible inhibition of the “rejuvenation” of complex I. A reduced mitochondrial membrane potential has been found alongside a reduction in mitochondrial complex I activity in the BRIN-BD11 rat clonal pancreatic β-cell line silenced for the *Ctns* gene [66], in the *Ctns*-knockout mouse model [29], in the HK-2 cell line silenced for the *CTNS* gene, and in epithelial proximal tubular cells from patients [53]. Additionally, a reduction in complex V F_1_F_O_-ATP synthase activity has been observed in cystinotic proximal tubular epithelial cells, which is associated with the downregulation of specific subunits such as “d” and OSCP that are involved in the dimerization and oligomerization of the complex [67]. This could be another cause of the reduced cellular ATP level. Interestingly, UCP-1 and UCP-2 protein levels were augmented in the gastrocnemius muscles and adipose tissues of *Ctns*^−/−^ mice compared to wild-type mice, suggesting increased energy expenditure associated with decreased ATP content, which contributes to muscle wasting and adipose tissue browning, respectively [68].

**Figure 2:**
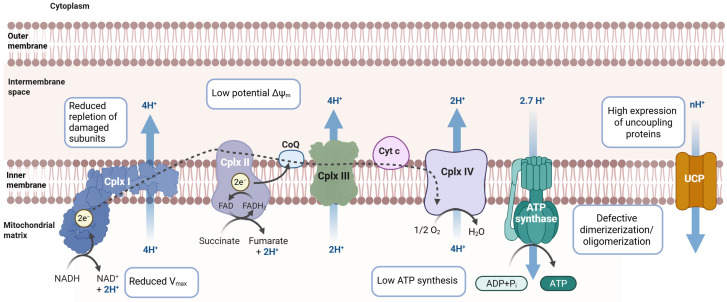
Mitochondrial energetics in cystinosis. Schematic representation of main defects involving respiratory chain complexes in models of cystinosis. Created in BioRender. Bellomo, F. (2026) https://BioRender.com/5l5uie1.

Another aspect of mitochondrial bioenergetics is nitric oxide (NO). NO plays an important role in renal pathophysiology; it is involved in fluid and electrolyte reabsorption by the proximal tubule. Most in vivo studies indicate that NO inhibits fluid and electrolyte reabsorption in the proximal tubule by inhibiting both the Na^+^/H^+^ exchange and the Na^+^, K^+^-ATPase activities. This condition is linked to renal Fanconi syndrome. Furthermore, NO has been found to be a potent inhibitor of cytochrome c oxidase of the mitochondrial RC [69]. Studies of the *Ctns* null PTECs line, which was isolated from *Ctns* knockout SV40T transgenic mice, revealed increased inducible nitric oxide synthase (iNOS) expression. This enzyme produces NO and nitrite/nitrate. It also reduces the expression and activity of Na^+^, K^+^-ATPase. These cells display depolarized mitochondria, a lower ATP content, altered nutrient metabolism, and are more sensitive to apoptosis due to their inhibitory effect on cytochrome *c* oxidase. Treating *Ctns* null PTECs with the iNOS-specific inhibitor 1400W recovered these parameters, which are associated with the mitigation of apoptosis. This explains, at least in part, the etiological factors of Fanconi syndrome in cystinosis. In a recent study, Berlingero et al. demonstrated that podocytes lacking *CTNS* exhibit impaired TCA cycle function and disrupted energy metabolism. This, in turn, resulted in reduced respiratory activity and elevated mitochondrial reactive oxygen species (ROS) levels [55] (Figure 2).

### 3.2. Mitophagy

The loss of cystinosin is associated with alterations in the endolysosomal compartment and proximal tubular cell dysfunction [70,71]. Endolysosomes capture and degrade worn-out intracellular components, including mitochondria, through a process called autophagy [72]. Starting with the observation that autophagy is required to protect proximal tubular cells from acute tubular injury by removing damaged mitochondria [73], an increase in altered mitochondria and SQSTM1/p62 receptors has been observed in urinary cells and kidney biopsies from cystinotic patients [74]. Furthermore, reduced basal autophagy flux has been found in various cystinotic cell lines, resulting in an increased number of mitochondria containing autophagosomes [33,75,76] associated with elevated LC3-II protein levels. Recently, studies in primary proximal tubular cells from *Ctns*-knockout mice and a zebrafish model demonstrated that lysosomal dysfunction leads to defects in the clearance of damaged mitochondria by autophagy [29]. Interestingly, as with mitochondrial RC activity, defects in autophagy have not been found in *Ctns*^−/−^ fibroblasts [33] likely because they rely on glycolysis rather than mitochondrial oxidative phosphorylation for energy. This aspect also highlights the tissue-specific role of cystinosin [8].

It has also been found that the cystinosin protein can physically interact with the molecular partners of the mammalian target of rapamycin complex 1 (mTORC1) [77,78]. The mTORC1 pathway modulates cellular metabolism, survival, proliferation, and autophagy [79,80]. Inhibiting the mTORC1 pathway with everolimus, a clinically used anticancer and immunosuppressant agent, activates autophagy [81,82] and abolishes the block of autophagic flux [83].

### 3.3. Mitochondrial Dynamics and Ultrastructure

Mitochondria are dynamic structures that undergo a process of dynamic structural changes, including fission and fusion, forming either fragmented or networked mitochondria, respectively, also depending on the energy status of the cell [84].

The process of mitochondrial fission plays a pivotal role in the formation of new mitochondria and the segregation of damaged mitochondria. In the event of impaired mitochondrial dynamics, defective mitochondria undergo selective elimination through a specialized form of autophagy, termed mitophagy. This process is initiated by signals that are either ubiquitin-independent or ubiquitin-dependent [85]. The process of fission is subject to regulation by the GTPase dynamin-related protein 1 (Drp-1), which is recruited from the cytosol to the mitochondrial outer membrane. In this location, Drp-1 binds to its protein receptors, including mitochondrial fission 1 (Fis-1), mitochondrial fission factor (MFF), and mitochondrial dynamics proteins, MID49 and MID51 [86,87]. According to recent research, *CTNS*^−/−^ PTECs exhibits a greater incidence of mitochondrial fragmentation, which is concomitant with diminished mitochondrial potential [54]. The condition is meticulously regulated and intimately linked to the quality control systems of the cell, such as the ubiquitin protease system (UPS) and the intra-mitochondrial proteolytic systems [88]. Despite the absence of alterations in Drp-1 protein levels and its PKA-dependent phosphorylation at Ser-637, as indicated by mitochondrial fragmentation [53], an increase in the expression of Fis-1 and the E3 ubiquitin ligase parkin, along with the ubiquitination of Mfn2, was observed in *CTNS*^−/−^ PTECs [54]. The results of the increase in parkin protein expression in cystinotic cells have become more significant considering the altered mitophagy observed in other studies [30,51,75]. In fact, parkin, a component of the PINK1/Parkin pathway, has been shown to promote mitophagy, a process that facilitates the elimination of damaged mitochondria. This, in turn, contributes to enhanced mitochondrial quality control, a critical aspect of cellular health and function. However, in the context of cystinosis, excessive mitophagy can be a cellular response to overwhelming damage. In cases where the lysosomal system is unable to adequately perform its function, this process can become counterproductive, resulting in a net loss of mitochondria.

The process of mitochondrial fusion is governed by a minimum of three regulatory fusion proteins, including the dynamin-related GTPase optic atrophy 1 (OPA1) and the dynamin-related GTPase mitofusins, Mfn1 and Mfn2 [89]. OPA1, an inner mitochondrial membrane protein, functions beyond mitochondrial fusion to regulate the stability of the mitochondrial RC complexes, the release of cytochrome c from mitochondrial cristae, and the maintenance of the architecture of mitochondrial cristae [90]. OPA1 activity is subject to rigorous regulation by post-translational modifications, including proteolytic processing and acetylation [91,92]. In fact, OPA1 undergoes a constitutive proteolytic process, during which the uncleaved long OPA1 (L-OPA) is converted into the cleaved shorter form of OPA1 (S-OPA). A variety of stress conditions, including apoptotic stimulation, have been shown to trigger the complete conversion of L-OPA1 into S-OPA1 [93]. The primary mitochondrial protease that regulates the proteolytic processing of OPA1 is OMA1 [94,95]. The total protein expression of OPA1 remained unchanged in cystinotic PTECs compared to control PTECs. However, an increase in S-OPA1, associated with an increase in the protease OMA1 activity, has been observed in *CTNS*^−/−^ PTECs [54]. It is noteworthy that, in addition to its function as a fusion protein, OPA1 modulates the restructuring of mitochondrial cristae. Specifically, OPA1 has been shown to form oligomers within the inner mitochondrial membrane, thereby maintaining the structural integrity of the cristae junctions [89]. During the process of apoptosis, the oligomers undergo a state of destabilization, thereby inducing the opening of the cristae and the subsequent dissipation of cytochrome c from the mitochondria. OPA1 oligomers were found to decrease in cystinotic cells. The association between the defect and the increase in cristae junction width was determined through TEM ultrastructural analyses [54].

Furthermore, ultrastructural analysis of mitochondria in PTECs, utilizing transmission electron microscopy (TEM), revealed the presence of smaller mitochondria in *CTNS*^−/−^ PTECs compared to *CTNS*^+/+^ PTECs. This observation is accompanied by a significant reduction in the number of cristae per mitochondrial section and an increase in cristae lumen width [54]. It is noteworthy that the mitochondrial ultrastructure observed in cystinotic cells is closely associated with the decrease in mitochondrial RC activity and the increased susceptibility to apoptosis [89,96].

**Figure 3:**
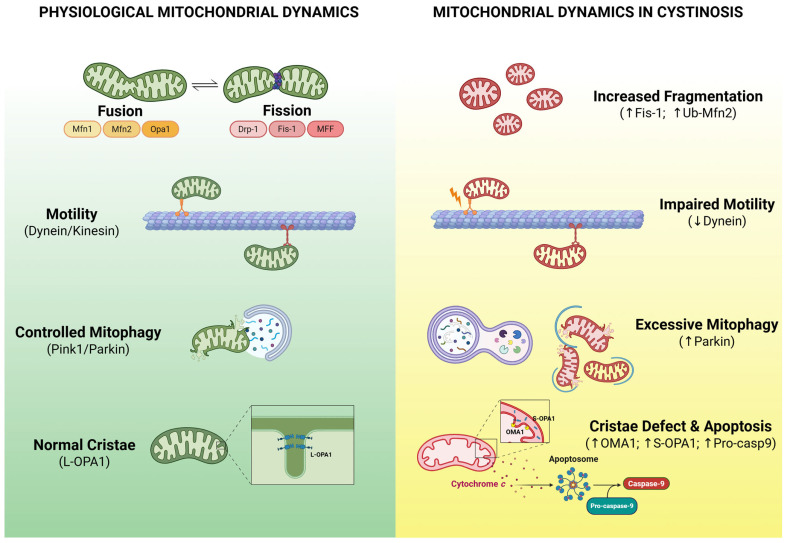
Mitochondrial Dynamics in Health and Cystinosis. In healthy subjects, mitochondrial fusion and fission maintain organelle shape and function. The process of fusion, facilitated by Mfn1/2 and OPA1, is crucial for maintaining network integrity and ensuring cristae stability. Conversely, the process of fission, driven by Drp-1 and its receptors (Fis-1, MFF), is essential for the segregation of damaged mitochondria for subsequent quality control. The dysregulation of these proteins has been demonstrated to induce mitochondrial fragmentation in cystinosis, in particular the upregulation of the Fis-1 and the ubiquitinated Mfn2 (Ub-Mfn2). The process of mitochondrial motility is contingent upon the function of microtubule-based motors, namely dynein and kinesin, which facilitate the distribution of mitochondria throughout the cell. This distribution ensures the proper positioning of these organelles for the efficient supply of energy and maintains a balance between the processes of fusion and fission. In *CTNS*^−/−^ cystinotic cells, reduced dynein (DYNC1LI2) expression has been shown to impair transport, resulting in stalled, clumped mitochondria and contributing to fragmentation and dysfunction. Autophagy is a process that maintains cellular health by removing damaged organelles and recycling nutrients. In healthy cells, the PINK1/Parkin pathway facilitates mitophagy, a process that selectively eliminates dysfunctional mitochondria. This process is crucial for maintaining energy balance and preventing oxidative stress. The loss of cystinosin has been demonstrated to disrupt endolysosomal function and impair autophagy in proximal tubular cells, associated with the increase of Parkin expression. This, in turn, has been shown to lead to the accumulation of damaged mitochondria. The process of mitochondrial fusion is dependent on the presence of OPA1 and mitofusins (Mfn1/2). The long form L-OPA1 is the uncleaved form of OPA1. In cystinosis, stress can trigger a cleavage process by the protease OMA1. In cystinosis, high OMA1 activity increases S-OPA1 levels and compromises mitochondrial structure. This process causes the cristae to become unstable and promotes the release of cytochrome c. This is associated with an increase in pro-caspase 9 expression and apoptosis. Created in BioRender. Bellomo, F. (2026) https://BioRender.com/l39flvx.

The process of mitochondrial motility is dependent on the function of microtubule-based motors, specifically dynein and kinesin, and is critical for maintaining a balance between fusion and fission [97]. Recent findings have indicated a decrease in the expression of the motor protein subunit dynein light intermediate chain DYNC1LI2 in the kidneys and fibroblasts of *Ctns*^−/−^ mice, as well as in the *CTNS* knockout human proximal tubule cell line [98]. The mechanistic role of DYNC1LI2 in mitochondrial transport remains to be fully elucidated. However, its reconstitution has been shown to restore vesicle/ER-lysosome trafficking, alleviate ER stress, and significantly mitigate mitochondrial fragmentation, membrane potential, and ER-mitochondrial associations in cystinotic cells [98]. The altered mitochondrial morphology has not been found in *CTNS*-silenced human induced pluripotent stem cells (iPSCs) [99], but this may be due to the more glycolytic metabolism of iPSCs than oxidative [100] (Figure 3).

### 3.4. The Mitochondrial–Inflammatory Axis

Mitochondria have been demonstrated to play a pivotal role in the progression of inflammation in cystinosis, thereby establishing a link between lysosomal cystine accumulation and subsequent immune activation. In the context of nephropathic cystinosis, mitochondrial dysfunction has been demonstrated to promote the generation of excess reactive oxygen species (ROS), which in turn amplifies inflammatory signaling pathways, thereby contributing to tissue injury. The upregulation of inflammatory mediators—including cytokines, inflammasome components, and macrophage-recruiting signals—interacts with mitochondrial stress, abnormal autophagy, and enhanced cell death, forming a self-reinforcing pathogenic loop.

This loop is now understood to be reinforced at the epigenetic level. Recent genome-wide analyses indicate that the cystinotic environment triggers extensive DNA hypermethylation, particularly in genes essential for proximal tubule physiology, effectively “locking” the cells into a dysfunctional state that can be partially reversed by demethylating agents [101]. Beyond the established NLRP3 pathway, the NLRP2 protein has emerged as a critical in vivo mediator of this process. The deletion of NLRP2 in *Ctns*^−/−^ models has been demonstrated to markedly delay the onset of Fanconi syndrome and reduce the expression of profibrotic markers and chemokines such as Cxcl1 and Saa1, thereby identifying it as a primary driver of the early inflammatory phase [102].

Furthermore, studies demonstrate that impaired cystinosin function alters lysosomal–mitochondrial crosstalk, leading to increased expression of inflammatory molecules such as galectin 3 and monocyte chemoattractant protein 1. These molecules drive macrophage infiltration and exacerbate renal inflammation [103].

Additionally, cystine crystals are potent activators of the NLRP3 inflammasome in macrophages, acting as a chronic, sterile inflammatory trigger. Recent evidence suggests this inflammatory state induces ‘trained immunity’, a form of innate immune memory characterized by epigenetic and metabolic reprogramming [104]. In the cystinotic kidney, resident macrophages functioning as crystal “custodians” may acquire a maladaptive memory phenotype. Once reprogrammed, these cells could perpetuate inflammation and drive fibrosis autonomously, independent of the concurrent crystal burden. Furthermore, because trained immunity necessitates a metabolic shift toward aerobic glycolysis, this process likely exacerbates the intrinsic energy metabolism defects associated with *CTNS* deficiency, creating a compounding metabolic insult.

Collectively, these findings underscore the pivotal role of mitochondria in integrating metabolic stress and inflammatory responses in cystinosis, thereby suggesting that targeting mitochondrial dysfunction, in conjunction with epigenetic modulators or NLRP2 inhibitors, may prove efficacious in mitigating chronic inflammation and decelerating kidney disease progression.

## 4. Impact of Cysteamine and Emerging Therapies on Cellular Energy Pathways

In contemporary clinical practice, cysteamine remains the only recognized agent employed in the management of cystinosis, with its mechanism of action specifically targeting the depletion of cystine. Cysteamine has been demonstrated to significantly delay the progression of kidney disease; however, it does not prevent the onset of renal Fanconi syndrome or halt the progression of the disease’s extrarenal manifestations. Nonetheless, a variety of experimental therapeutic approaches are currently underway, encompassing the use of small molecules, enzymes, gene and cell-based therapies, and other potential treatments that are in various stages of development [105,106,107,108,109,110,111].

### 4.1. Cysteamine

Cysteamine exerts significant effects on cellular energy metabolism, primarily through modulation of mitochondrial function. In a pre-clinical study, cysteamine bitartrate was used to treat mitochondrial RC disease in worms, zebrafish, and human fibroblasts. High doses caused toxicity via hydrogen peroxide, while lower doses showed limited benefits. In worms, treatment reduced oxidative stress and improved mitochondrial function, though it did not improve survival. Human fibroblasts exhibited broad metabolic recovery. Pre-treatment with cysteamine in zebrafish prevented brain death and neuromuscular defects caused by RC inhibitors. This highlights cysteamine’s neuroprotective potential through enhancement of the oxidative stress response in RC disorders such as Leigh syndrome and MELAS.

At low micromolar concentrations, cysteamine has been shown to enhance ATP levels, restore oxidative phosphorylation, improve mitochondrial membrane potential, and maintain mitochondrial structure in cystinotic proximal tubular cell cultures [52,53,54]. Similarly, improved membrane potential and oxidative phosphorylation have been observed in C. elegans complex I mutants (NDUFS2 orthologue) and human fibroblasts of Leigh syndrome patients without significant alterations in glutathione levels, indicating the presence of alternative antioxidant or metabolic pathways [112]. Cysteamine has also been demonstrated to influence metabolite flux, notably increasing aspartate availability, and reducing ROS under stress conditions. However, its therapeutic window is narrow: while low doses confer bioenergetic and antioxidant benefits, millimolar concentrations can induce oxidative stress and cellular toxicity. Cysteamine demonstrated an inability to improve alterations in the tricarboxylic acid (TCA) cycle or energy metabolites, as well as an absence of oxidative stress, in podocytes from *CTNS*^−/−^ patients [55]. In addition, the increased cristae junction width observed in cystinotic renal cell cultures showed a non-reversible response to cysteamine treatment, paralleling the reduction in OPA1 oligomerization [54]. Finally, the administration of cysteamine resulted in the restoration of mitochondrial cAMP levels in PTECs lacking *CTNS*, concomitant with enhanced SIRT3 levels [53]. Mitochondrial cAMP is produced by soluble adenylyl cyclase and regulates localized signaling independent of plasma membrane GPCRs. Specifically, mitochondrial cAMP activates PKA within the mitochondrial matrix, leading to the phosphorylation of components of the electron transport chain, thereby enhancing ATP production [113]. SIRT3 has been identified as a pivotal mitochondrial deacetylase, involved in crucial cellular processes such as enhancing electron transport, TCA flux, antioxidant defenses, and mitophagy [114].

### 4.2. Flavonoids

Flavonoids, a diverse group of plant-derived polyphenols, have attracted significant attention for their ability to modulate mitochondrial function and protect against cellular stress [115]. Two studies investigated the molecular mechanisms of flavonoid compounds in cystinosis models through complementary experimental approaches. Luteolin was identified through high-throughput screening and subsequently tested in the *Ctns*^−/−^ mouse model. The results demonstrated an improvement in the autophagy-lysosome degradative pathway, as evidenced by reduced p62/SQSTM1 levels measured by in-cell ELISA. Additionally, enhanced megalin expression was observed, along with antioxidant and antiapoptotic properties [31,110]. Genistein, which exhibited efficacy in a 14-month preclinical trial conducted in *Ctns*^−/−^ mice, demonstrated a reduction in kidney cystine concentrations, cystine crystal formation, and LAMP1-positive structures while preserving parenchymal architecture. It has been demonstrated that both compounds act through cysteamine-independent mechanisms, targeting cellular pathways beyond lysosomal cystine accumulation [111]. It is noteworthy that nutraceutical compounds, such as genistein and luteolin, have been observed to enhance cAMP levels [116,117]. Flavonoids have been reported to modulate the endogenous levels of antioxidants, including superoxide dismutase, catalase, and glutathione peroxidase, as well as the activity of enzymes responsible for glutathione synthesis, such as glutathione reductase [102,103]. In addition, flavonoids have been shown to stimulate mitochondrial biogenesis, which is an adaptive cellular response to various stressors. This stimulation occurs through the upregulation or activation of PGC-1α [118]. Furthermore, flavonoids have been demonstrated to stimulate a series of transcription factors, including NRF1 and NRF2, which play important roles in regulating metabolism-related genes [105,106]. Specifically, genistein has been observed to enhance the activating phosphorylation of Src (Tyr416) and the phosphorylation of acetyl-CoA carboxylase (ACC), a substrate of AMPK and a marker of AMPK activation [119]. Analogously, luteolin induces AMPK phosphorylation, which increases the activity of SIRT1, which in turn activates PGC1α by deacetylating it and increasing the phosphorylation of ACC.

### 4.3. MitoQ, mitoTEMPO and Other Compounds Selected in Drug Repositioning Studies

The mitochondria-targeted antioxidants mitoquinone (MitoQ) and mitoTEMPO are, respectively, the derivatives of mitochondrial quinolone and a superoxide dismutase mimetic that has superoxide and alkyl radical scavenging properties. MitoQ treatment has been shown to increase PGC1α expression and prevent mitochondrial membrane depolarization, reduce free radicals, and increase cytochrome oxidase activity [120]. MitoTEMPO treatment has been shown to attenuate mitochondrial oxidative damage by specifically reducing mitochondrial superoxide anion levels [121].

Recent findings indicate that the presence of swan-neck lesions in the renal proximal tubule associated with cystinosis is indicative of an adaptation to oxidative stress. These lesions have been observed to be associated with the loss of mitochondria and an increase in mitochondrial anion superoxide production. The administration of MitoQ to *Ctns*^−/−^ mice at one month of age or three months of age, continuing until six months of age, resulted in the preservation of the integrity of the glomerulotubular junctions and the deferral of the onset of the so-called swan-neck lesions. However, this intervention did not lead to the rescue of low-molecular-weight proteinuria [122].

Treatment of *Ctns*^−/−^ mouse PTECs for 24 h with mitoTEMPO has been demonstrated to promote the integrity of tight junctions and the differentiation and endocytic uptake capacity of these cells [22]. In preclinical studies investigating mitoTEMPO in immortalized patient-derived podocytes and zebrafish larvae models, the combination therapy with cysteamine has been shown to improve clinical outcomes. Indeed, cysteamine, when administered as a standalone agent, demonstrated no significant impact. However, when administered concomitantly with the mitochondrial O_2_^•−^ scavenger MitoTEMPO, there was a substantial reduction in the ROS-induced lipid peroxidation, improved cell adhesion and reduced podocyte permeability and a significant reduction in proteinuria in zebrafish [55].

A total of five compounds were identified as effective in reducing cystine content and apoptosis in human *CTNS*^−/−^ PTECs. This identification was made through a combination of high-throughput and high-content drug screenings [123]. The subsequent computational analysis of these selected drugs, namely alexidine dihydrochloride, beta-escin, digoxin, disulfiram, and fluspirilene, demonstrated a shared mechanism of action in cystinotic cells, predominantly involving the regulation of metabolic processes, response to stress, RTK signaling pathway, cell–cell adhesion molecules, and mitoribosomes [123]. However, subsequent studies performed on mouse and zebrafish cystinosis models revealed that prolonged exposure to the selected drug disulfiram, even if was effective in cystine reduction, resulted in toxicity in both animal models [124]. The pleiotropic effects of disulfiram on mitochondria are of particular interest. Indeed, it has been demonstrated that this compound can rescue mitochondrial defects in disease models such as Barth syndrome or MELAS, by enhancing oxidative phosphorylation, improving ATP synthase, respiratory complexes III/IV, cardiolipin remodeling, and protein synthesis inside mitochondria [125]. However, disulfiram can also trigger mitochondrial dysfunction by forming a complex with copper ions (DSF-Cu) within cells. This complex leads to a cascade of events, including the accumulation of ROS and the disruption of key mitochondrial functions [126]. Consequently, the outcomes observed in animal models of cystinosis were contingent upon the cellular context, copper levels, and redox status, which could have contributed to the toxic effect of disulfiram.

### 4.4. Novel Treatment Strategies That Could Affect Mitochondrial Function

An increase in the protein content of UCPs was observed in the adipose tissues and muscles of *Ctns*^−/−^ mice compared with the control group. Conversely, a decrease in ATP content was observed in adipose tissues and muscles of *Ctns*^−/−^ mice in comparison with the control group. The presence of vitamin D deficiency has been demonstrated to have a detrimental effect on adipose tissue and muscle metabolism. As demonstrated by Cheung et al., the repletion of 25(OH)D_3_ and 1,25(OH)_2_D_3_ was found to attenuate the perturbations of uncoupling protein (UCP) and adenosine triphosphate (ATP) contents in adipose tissues and muscles in *Ctns*^−/−^ mice [127]. It is noteworthy that vitamin D has been observed to modulate the expression of mitofusin-1/2 (Mfn1/2), OPA1, and DRP1, as well as the shape of mitochondrial cristae [128,129].

Another molecule, designated 1400W, has been shown to reduce nitric oxide production by inhibiting iNOS. This molecule has demonstrated a remarkable efficacy in counteracting the effects of Fanconi syndrome. It is imperative to acknowledge that NO, which has been demonstrated to impede both Na^+^/H^+^ exchange and Na^+^, K^+^-ATPase activity, concurrently functions as a robust inhibitor of cytochrome c oxidase within the mitochondrial RC [130]. This phenomenon has the potential to result in depolarized mitochondria and diminished ATP levels, which, in turn, could lead to an escalation in apoptosis. In a study of *Ctns*-null PTECs, the administration of 1400W was found to restore normal mitochondrial function, improve ATP content, normalize nutrient metabolism, and reduce apoptosis [131].

Recent findings indicate that ketogenic diets (KD), which shift energy metabolism from glucose to ketone bodies, significantly preserve kidney function in mouse models of nephropathic cystinosis. This preservation occurs even when the diets are initiated after the onset of the disease. The KD prevents Fanconi syndrome-related proteinuria, glycosuria, polyuria, inflammation, and fibrosis [109,132]. To date, the tissue-specific effects of KD have been demonstrated by its ability to enhance mitochondrial function and biogenesis in certain tissues, thereby supporting its therapeutic application in neurological and metabolic disorders. Indeed, KD has been observed to upregulate key regulators of mitochondrial biogenesis and function, including PGC1α, SIRT3, and UCP2. This upregulation results in increased mitochondrial mass and enhanced bioenergetics, particularly in neurons and skeletal muscle [133,134].

As illustrated in Table 1, a comparative analysis is presented, encompassing the mechanisms of action and the effect on mitochondrial parameters.

## 5. Conclusions

**Figure 4:**
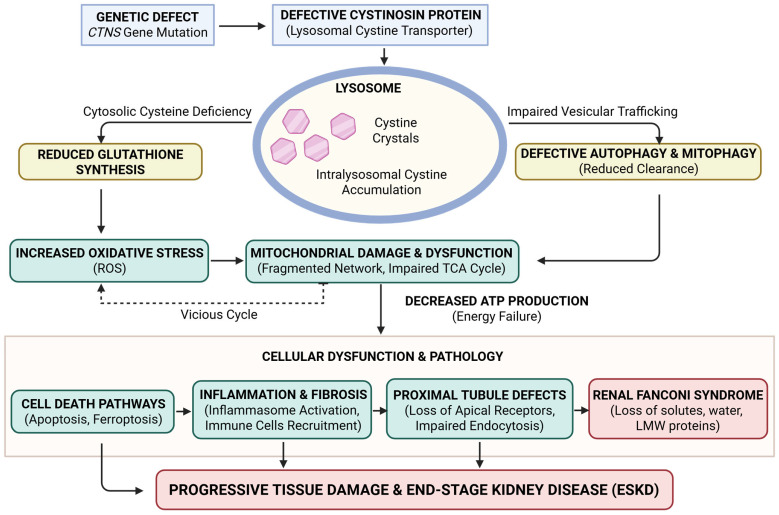
Pathways Leading to Cellular Dysfunction in Cystinosis. Mutations in the CTNS gene result in a defective or absent cystinosin protein, the primary lysosomal cystine transporter. This defect leads to the hallmark intra-lysosomal accumulation of cystine and the formation of cystine crystals. The resulting cellular pathology is driven by two main arms: on left branch, cytosolic cysteine deficiency impairs glutathione synthesis, leading to increased reactive oxygen species (ROS) and oxidative stress. On right branch, impaired vesicular trafficking results in defective autophagy and mitophagy, reducing the clearance of damaged organelles. These pathways converge to cause significant mitochondrial damage and dysfunction, characterized by a fragmented mitochondrial network and an impaired TCA cycle. This creates a “vicious cycle” of oxidative stress and energy failure (decreased ATP production). The cumulative cellular dysfunction triggers cell death pathways (apoptosis and ferroptosis), chronic inflammation, and fibrosis. In the kidney, these processes manifest as proximal tubule defects and Renal Fanconi Syndrome, ultimately culminating in progressive tissue damage and End-Stage Kidney Disease (ESKD). Abbreviation: ESKD: End-stage kidney disease; LMW: Low-molecular-weight; ROS: Reactive oxygen species; TCA: Tricarboxylic acid cycle. Created in BioRender. Bellomo, F. (2026) https://BioRender.com/caur1ds.

Cystinosis provides a compelling example of how lysosomal dysfunction can lead to a significant cellular energy failure, with mitochondria playing a pivotal role in the progression of the disease. The available evidence indicates that cystine accumulation disrupts mitochondrial morphology, impairs oxidative phosphorylation, and increases oxidative stress. This, in turn, compromises proximal tubular cell viability and systemic energy balance (see Figure 4). These findings establish mitochondria not only as downstream targets but also as promising therapeutic targets. Consequently, strategies aimed at restoring mitochondrial function—through antioxidants, metabolic modulation, or gene-based approaches—may enhance the benefits of cysteamine.

Elucidation of the mechanisms by which lysosomal cystine overload modifies mitochondrial signaling, dynamics, and inter-organelle communication contributes to a more profound comprehension of the pathophysiology underlying cystinosis. This enhanced knowledge facilitates the development of novel therapeutic interventions, including gene therapy, mRNA-based approaches, and stem cell-based strategies. These methodologies aim to either restore cystinosin function or to rectify downstream mitochondrial defects, underscoring the significance of mechanistic research in propelling precision therapies.

Further research should delineate the roles of mitochondrial stress responses, mitophagy, and organelle crosstalk while developing reliable biomarkers of mitochondrial dysfunction for early diagnosis and monitoring. The development of novel therapeutic interventions will be contingent upon the exploration of mitochondria-targeted compounds and combination regimens that have been validated in robust preclinical models. By addressing these priorities, research can transition from a focus on managing symptoms to developing strategies that address the underlying bioenergetic deficits driving cystinosis progression.

## Figures and Tables

**Table 1:** Mechanism of action and effect on mitochondrial parameters of candidate therapeutic agents.

Therapeutic Agent	Mechanism of Action	Effect on Mitochondrial Parameters		Key Observations/Limitations
Cysteamine	Restores mitochondrial cAMP (via soluble adenylyl cyclase), activating PKA and increasing SIRT3 levels.	Enhances ATP levels, restores OXPHOS, improves membrane potential, maintains structure. Phosphorylation of ETC components enhances ATP production. SIRT3 enhances TCA flux and antioxidant defenses. Does not correct TCA cycle defects or energy metabolites in podocytes.		Low doses are beneficial; high doses cause H_2_O_2_ toxicity. Cannot reverse increased cristae junction width or OPA1 oligomerization defects.
Flavonoids (Genistein & Luteolin)	Luteolin induces AMPK phosphorylation → SIRT1 activation → PGC-1α deacetylation. Genistein enhances Src and ACC phosphorylation. Increases cAMP levels; activates NRF1/NRF2.	Stimulates mitochondrial biogenesis via PGC-1α upregulation. Modulates SOD, Catalase, and Glutathione Peroxidase. Improvements in autophagy-lysosome pathways (reduced p62).		Acts via cysteamine-independent mechanisms. Preserves kidney architecture and reduces cystine crystals.
MitoQ	Mitochondria-targeted antioxidant, derivative of mitochondrial quinone.	Increases cytochrome oxidase activity and PGC-1α expression. Prevents membrane depolarization and reduces free radicals.		Delays onset of “swan-neck” lesions (an adaptation to oxidative stress) but does not rescue proteinuria.
MitoTEMPO	Acts as superoxide and alkyl radical scavenger specifically in mitochondria.	Specifically reduces mitochondrial superoxide anions. Combined with cysteamine, reduces lipid peroxidation.		Promotes tight junction integrity. Effective in combination with cysteamine where cysteamine alone failed (e.g., in zebrafish podocytes).
Disulfiram	Inhibitor of aldehyde dehydrogenase, selected via high-content screening, with copper-binding properties.	In other diseases (MELAS/Barth), it rescues ETC complexes III/IV and ATP synthase. In cystinosis forms a toxic complex with Copper (DSF-Cu) causing ROS accumulation and mitochondrial disruption.		Although effective at reducing cystine, prolonged exposure caused toxicity in animal models due to redox status and copper levels.
Vitamin D (1,25(OH)_2_D_3_)	Replenishes deficiency common in cystinosis.	Modulates expression of fusion/fission proteins (Mfn1/2, OPA1, DRP1) and cristae shape. Attenuates perturbations in UCP and restores ATP content in muscle/adipose tissue.		Critical for addressing muscle wasting and adipose tissue browning in *Ctns*^−/−^ models.
1400W	Inhibits inducible Nitric Oxide Synthase, reducing NO production.	Prevents NO-mediated inhibition of Mitochondrial Complex IV. Restores ATP content, prevents mitochondrial depolarization, and reduces apoptosis.		Counteracts Fanconi syndrome features by restoring Na^+^/K^+^-ATPase activity (which is inhibited by NO).
Ketogenic Diet	Shifts energy metabolism from glucose to ketone bodies.	Modulates potentially PGC-1α, SIRT3, and UCP2.		Preserves kidney function; prevents inflammation and fibrosis

## Data Availability

No new data were created or analyzed in this study. Data sharing is not applicable to this article.

## Abbreviations

The following abbreviations are used in this manuscript:

ATP	Adenosine Triphosphate
cAMP	3′,5′-Cyclic Adenosine Monophosphate
CDME	Cystine Dimethyl Ester
CMA	Chaperone-Mediated Autophagy
DMT1	Divalent Metal Transporter 1
Drp-1	Dynamin-Related Protein 1
ER	Endoplasmic Reticulum
Fis-1	Mitochondrial Fission 1
iNOS	inducible Nitric Oxide Synthase
LSD	Lysosomal Storage Disease
MCSs	Membrane Contact Sites
MFF	Mitochondrial Fission Factor
Mfn2	Mitofusin-2
MID	Mitochondrial Dynamics Protein
MTOC	Microtubule-Organizing Center
mTOR	mechanistic Target of Rapamycin
mTORC1	Mammalian Target Of Rapamycin Complex 1
NO	Nitric Oxide
OMA1	OMA1 Zinc Metallopeptidase
OMIM	Online Mendelian Inheritance in Man
OPA1	Optic Atrophy Protein 1 (Mitochondrial Dynamin-Like GTPase)
OSCP	Oligomycin Sensitivity-Conferring Protein
OXPHOS	Oxidative Phosphorylation System
RILP	Rab Interacting Lysosomal Protein
SQSTM1	Sequestosome-1
STARD3	StAR Related Lipid Transfer Domain Containing 3
TRPML1	Transient Receptor Potential Mucolipin 1
UCP	Uncoupling Protein
Vps13	Vacuolar protein sorting 13
